# Adeno-Associated Virus Rep Represses the Human Integration Site Promoter by Two Pathways That Are Similar to Those Required for the Regulation of the Viral p5 Promoter

**DOI:** 10.1128/JVI.00412-14

**Published:** 2014-08

**Authors:** Nathalie Dutheil, Sarah C. Smith, Leticia Agúndez, Zoé I. Vincent-Mistiaen, Carlos R. Escalante, R. Michael Linden, Els Henckaerts

**Affiliations:** Department of Infectious Diseases, King's College London School of Medicine, London, United Kingdom

## Abstract

Adeno-associated virus serotype 2 (AAV2) can efficiently replicate in cells that have been infected with helper viruses, such as adenovirus or herpesvirus. However, in the absence of helper virus infection, AAV2 establishes latency by integrating its genome site specifically into *PPP1R12C*, a gene located on chromosome 19. This integration target site falls into one of the most gene-dense regions of the human genome, thus inviting the question as to whether the virus has evolved mechanisms to control this complex transcriptional environment in order to facilitate integration, maintain an apparently innocuous latency, and/or establish conditions that are conducive to the rescue of the integrated viral genome. The viral replication (Rep) proteins control and direct every known aspect of the viral life cycle and have been shown to tightly control all AAV2 promoters. In addition, a number of heterologous promoters are repressed by the AAV2 Rep proteins. Here, we demonstrate that Rep proteins efficiently repress expression from the target site *PPP1R12C* promoter. We find evidence that this repression employs mechanisms similar to those described for Rep-mediated AAV2 p5 promoter regulation. Furthermore, we show that the repression of the cellular target site promoter is based on two distinct mechanisms, one relying on the presence of a functional Rep binding motif within the 5′ untranslated region (UTR) of *PPP1R12C*, whereas the second pathway requires only an intact nucleoside triphosphate (NTP) binding site within the Rep proteins, indicating the possible reliance of this pathway on interactions of the Rep proteins with cellular proteins that mediate or regulate cellular transcription.

**IMPORTANCE** The observation that repression of transcription from the adeno-associated virus serotype 2 (AAV2) p5 and integration target site promoters is mediated by shared mechanisms highlights the possible coevolution of virus and host and could lead to the identification of host factors that the virus exploits to navigate its life cycle.

## INTRODUCTION

Adeno-associated virus serotype 2 (AAV2) is a human DNA virus that is dependent upon a number of factors provided by helper viruses in order to replicate efficiently (1–4). In the absence of helper factors, AAV2 has the ability to establish latency by site-specifically integrating its genome into chromosome 19 (5, 6). A complex interplay between host cellular, AAV2, and helper virus proteins leads to the tight regulation of a life cycle that is unique among eukaryotic viruses. The nonstructural proteins Rep78, Rep68, Rep52, and Rep40, encoded by the AAV2 *rep* genes, play a major role in orchestrating the different aspects of the AAV2 life cycle. The large Rep proteins, Rep78 and Rep68, transcribed from the p5 promoter, are multifunctional proteins with DNA binding, endonuclease, and helicase activities that control replication, integration, and transcription (reviewed in reference 7). The small Rep proteins, transcribed from the p19 promoter, share the nucleoside triphosphatase (NTPase) and helicase activity with the large Rep proteins and are required for efficient packaging of the viral particles (8). The Rep proteins have a distinctive autoregulatory role in that they control p5 and p19 transcription as well as p40-controlled transcription of the structural proteins. In the presence of helper virus, repression of the p5 promoter by Rep and cellular factors YY1 and MLTF is lifted (9–11), which leads to transactivation of transcription from all three promoters (12) but is regulated so that p40 transcript levels are higher than the p5 and p19 transcript levels (13). Sequences within, as well as outside, the viral promoter regions have been shown to be involved in Rep activation (14). Interestingly, during productive infection, the Rep proteins can mediate both activation and repression of transcription (15). More specifically, the large Rep proteins activate transcription from the p5 promoter through binding to the Rep binding site (RBS) present in the inverted terminal repeat (ITR) and mediate p5 repression by binding to the RBS in the p5 promoter. This repression can be partially lifted by the small Rep proteins and contributes to an autoregulatory loop, which maintains constant ratios of the p5 and p19 transcripts (16).

In the absence of helper virus, p5, p19, and p40 transcription is significantly reduced, leading to minute levels of Rep protein expression (17–19). Rep-mediated repression of p5 transcripts appears to be dependent on the NTP-binding motif present in the central domain of the Rep proteins, as well as the presence of an intact RBS motif in the p5 promoter (20). In contrast, Rep-mediated repression of the p19 promoter requires only the NTP-binding motif of Rep (19). Transcriptional repression by Rep is not exclusive to AAV2 promoters but has also been observed for heterologous promoters, such as the HIV long terminal repeat (LTR), the human papillomavirus 18 (HPV18) upstream regulatory region (URR), and the major late transcription promoter of adenovirus (AdMLP), and is dependent on the Rep NTP-binding domain (21, 22). Rep also affects the expression of cellular genes, such as those encoding c-myc and c-sis/platelet-derived growth factor B (23–25); however, the significance of Rep-mediated regulation of these promoters in the context of AAV2's life cycle has yet to be established.

AAV2 has the ability to site-specifically integrate its genome into a gene, *PPP1R12C*, that encodes a protein that is thought to be a component of the regulatory subunit of myosin light chain phosphatase (26). Analysis of the 5′ untranslated region (UTR) of this gene led to the observation that the minimal promoter region of *PPP1R12C* and the p5 promoter have two important *cis*-regulatory elements in common, namely, the RBS and YY1 sites (27). This observation, together with the fact that AAV2 establishes latency by integrating into a ubiquitously transcribed locus, provoked the question of whether AAV2 has evolved the ability to control transcription of the AAV2 integration target locus in order to aid the establishment of latency and secure viral rescue.

We demonstrate here that Rep proteins efficiently repress expression from the target site *PPP1R12C* promoter, employing mechanisms similar to those described for Rep-mediated viral p5 promoter regulation. We provide evidence that the observed repression is based on two distinct mechanisms: one relies on the presence of a functional Rep binding motif within the 5′ UTR of *PPP1R12C*, whereas the second pathway requires only an intact NTP-binding site within the Rep proteins, possibly indicating the reliance of this pathway on interactions of the Rep proteins with factors of or associated with the cellular transcription machinery. We propose that the p5 promoter has coevolved with the host *PPP1R12C* promoter, thereby ensuring the possibility that Rep can control the expression at the viral integration site.

## MATERIALS AND METHODS

### Cell lines and viruses.

HEK-293T/17 human embryonic kidney cells (ATCC CRL-11268) and HeLa human cervical epithelial cells (ATCC CCL-2) were cultured in Dulbecco's modified Eagle's medium (DMEM) (Invitrogen) supplemented with 10% fetal bovine serum (FBS) (Invitrogen).

AAV2 and human adenovirus type 5 (Ad5) were produced and purified as previously described (28).

### Infections.

Cells were infected in 60-mm plates at 70% confluence by adding increasing amounts of wild-type (wt) AAV2 (multiplicity of infection [MOI], 1 to 10^4^ infectious units per cell) to 1 ml of DMEM and 10% FBS. After 2 h of incubation at 37°C, 293T and HeLa cells were coinfected with adenovirus type 5 at an MOI of 5 and 10 PFU, respectively. After 1 h of incubation at 37°C, the inoculum was removed, and 3 ml of fresh medium was added to the cells.

### Reporter constructs.

To increase the sensitivity of the reporter construct, we replaced DsRed2.1 with mCherry in the previously used −332/+94 plasmid (pND26). The plasmid contains the *PPP1R12C* promoter region from nucleotides (nt) −332 to +94 relative to the transcription start site (27).

The PPP1R12Cp-mCherry (pND203) reporter construct was cloned in two steps. The mCherry open reading frame (ORF) was first amplified by PCR using ND381 (5′ GGATCCCACAACCATGGTGAGCAAGGGC-3′) and ND382 (5′-GCGGCCGCTACTTGTACAGCT-3′) as primers and pTW149 as the template (28). The resulting PCR product was cloned into pCR2.1 (Invitrogen) to create pND202. To generate the PPP1R12Cp-mCherry (pND203) and p5-mCherry (pND208) reporter constructs, the mCherry sequence linked to a BamHI/NotI fragment from pND202 was subcloned into the BamHI/NotI-digested pND26 and p5 vectors (pND85), respectively (27). The p5-mCherry construct contains the p5 promoter region (nt 190 to nt 310 relative to the AAV2 sequence) described by Cheung et al. (29).

Mutations within the *PPP1R12C* RBS were introduced by inserting annealed primers into the BamHI/SmaI sites of the pND23 plasmid (the *PPP1R12C* promoter region from nt −332 to +20 cloned into pBluescript II SK [Fermentas]) (27). The resulting plasmid, pND31 (the *PPP1R12C* promoter region from nt −332 to +94), was digested with BamHI and HindIII, and the *PPP1R12C* promoter fragment containing the mutated RBS motif was blunt ended prior to ligation into the SmaI site of the pDsRed2.1 vector (Clontech) to create plasmid pND34 (PPP1R12Cp-RBS*-DsRed2.1). To generate the PPP1R12Cp-RBS*-mCherry reporter construct (pND212), the mCherry-containing BamHI/NotI fragment from pND202 was subcloned into the BamHI/NotI sites of the pND34 plasmid. The same mutation was introduced into the p5 RBS by site-directed mutagenesis on the pND84 (p5-pCR2.1) template (27) to create pND235. To generate the p5-RBS*-mCherry construct (pND236), the SmaI fragment containing the p5 promoter region from pND235 was inserted into the SmaI sites of pND208.

To generate the pEGFP-PPP1R12Cp-mcherry bidirectional promoter construct (pND238), the blunt-ended AflII/MscI enhanced green fluorescent protein (EGFP)-encoding fragment of pIRES2-EGFP (Clontech) was subcloned into the blunt-ended EcoRI site of pND203.

### Rep-expressing constructs.

All Rep-expressing constructs were cloned into the pIRES2-EGFP vector. To generate the Rep78-expressing plasmid (pR78-IRES2-EGFP), the Rep78-encoding DraI/SphI fragment from plasmid pHis-Rep78 (30) was first ligated to annealed adaptors and inserted between the SmaI and NheI sites of the pIRES2-EGFP vector. To generate pR78Y156F-IRES2-EGFP, the SacII/SalI fragment containing the Y156F mutation from plasmid pHis-Rep68Y156F (30) was cloned into the SacII/SalI-digested pR78-IRES2-EGFP vector. To generate pRep68Y156F-IRES2-EGFP (pND21), the BamHI fragment of pHis-Rep68 (31) containing the C-terminal region of Rep68 was inserted between the BamHI sites of pR78Y156F-IRES2-EGFP. The initiation methionine of Rep52 and Rep40 was mutated to a glycine (M225G) by site-directed mutagenesis. The resulting PCR fragment was cloned into the pCR2.1 vector to generate pND102. The SacI/SalI fragment containing the M225G mutation from pND102 was inserted into the corresponding SacI/SalI fragment of pRep78Y156F-IRES2-EGFP and pND21 to generate pR78Y156F/M225G-IRES2-EGFP (pND105) and pR68Y156F/M225G-IRES2-EGFP (pND104), respectively.

The 5′ ends of Rep52 and Rep40 were generated by PCR on a pAV2 template with primers ND144 (5′-GATATCGCACAACATGGAGCTGGTCGGG-3′) and ND67 (5′-CATCCGGTCTTGCAACGGCTGC-3′), and the resulting PCR product was subcloned into the pCR2.1 cloning vector to create pND20. pRep52-IRES2-EGFP (pND22) was constructed by inserting the 5′ end of Rep52 containing the EcoRV/SalI fragment from pND20 into the EcoRV/SalI sites of pRep78Y156F-IRES2-EGFP. To generate pRep40-IRES2-EGFP (pND56), the Rep40 3′-end BamHI fragment from pND21 was inserted into the BamHI sites of pND22.

One, two, or three copies of the simian virus 40 (SV40) large T antigen nuclear localization signal (NLS), PKKKRKV, were added in frame to the 3′ end of the N208 open reading frame. PCR fragments amplified on pRep78Y156F-IRES2-EGFP with primers containing the NLS sequences were subcloned into pCR2.1 to generate pND59 (pN208Y156F-NLS1) and pND75 (pN208Y156F-NLS2). pND81 (pN208Y156F-NLS3) was generated by PCR on a pND75 template. To generate pN208Y156F-NLS_1_-IRES2-EGFP (pND62), pN208Y156F-NLS_2_-IRES2-EGFP (pND82#2), and pN208Y156F-NLS_3_-IRES2-EGFP (pND82#3), the PstI/BamHI fragment containing the 3′ end of N208 from pND59, pND75, and pND81 was inserted into the corresponding PstI/BamHI sites of pRep78Y156F-IRES2-EGFP.

To generate pRep68Y156F/K340H-IRES2-EGFP (pND25), the SacI/AccI fragment of pHis-Rep68K340H (32, 33), containing the K340H mutation, was inserted between the SacI/AccI sites of pND21. pRep68Y156F/M225G/K340H-IRES2-EGFP (pND140) and pRep40/K116H-IRES2-EGFP (pND80) were generated by inserting the BamHI fragment from pND25, containing the K340H mutation, into the corresponding BamHI fragments from pND104 and pND56. pRep78Y156F/M225G/K340H-IRES2-EGFP (pND146) and pRep52K116H-IRES2-EGFP (pND143) were generated by inserting a fragment containing the K340H mutation from pND140 (SacI/AccI) and pND80 (EcoRV/SalI) into the corresponding sites of pRep78Y156F-IRES2-EGFP.

To delete the internal ribosome entry site 2 (IRES2)-EGFP sequence from Rep-expressing vectors and generate pΔIRES2-EGFP (pND216), the plasmid pIRES2-EGFP was digested with BamHI and NotI, and the overhangs were blunt ended with the Klenow DNA polymerase prior to religation. The Rep78 and Rep68 ORFs were amplified by PCR from the pND105 and pND104 plasmids using the SmaI-containing primers ND387 (5′-CCCGGGATATCGCACAACATGCCGGGG-3′) and ND388 (5′-CCCGGGTTATTGTTCAAAGATGCAGTCATCCAAATC-3′), and ND387 and ND389 (5-CCCGGGTCAGAGAGAGTGTCCTCGAGC-3′), respectively. The resulting PCR fragments were subcloned into the PCR2.1 cloning vector to generate pND221 (Rep78Y156F/M225G-pCR2.1) and pND220 (Rep68Y156F/M225G-pCR2.1). pRep78Y156F/M225G-ΔEGFP (pND227) and pRep68Y156F/M225G-ΔEGFP (pND226) were generated by subcloning the Rep-encoding SmaI fragment from pND221 and pND220 into the SmaI site of pND216. pRep52-ΔEGFP (pND230) and pRep40-ΔEGFP (pND229) were generated by inserting the 5′ ends of the Rep52 and Rep40 EcoRV/BstEII fragments from pND22 and pND56 into the EcoRV/BstEII sites from pND227 and pND226, respectively. Plasmids pRep78Y156F/M225G/K340H-ΔEGFP (pND231), pRep68Y156F/M225G/K340H-ΔEGFP (pND232), pRep52K116H-ΔEGFP (pND233), and pRep40K116H-ΔEGFP (pND234) were generated by inserting the EcoRV/BstEII fragments containing the K340H mutation from plasmids pND146, pND140, pND143, and pND80 into the corresponding EcoRV/BstEII sites of plasmids pND227, pND226, pND230, and pND229. All the vectors were sequenced.

### Transient transfections.

Transient transfections were performed in 60-mm plates using Lipofectamine 2000 according to the manufacturer's instructions (Invitrogen). At 70% confluence, 293T cells were transfected with 4.5 μg of reporter plasmid and 9 μg of Rep-expressing plasmid. Cells were harvested 48 h posttransfection and assayed for plasmid DNA uptake and RNA and protein levels.

### Plasmid uptake determination.

The transfected cells were lysed in 0.2 M NaOH and 10 mM EDTA, boiled for 15 min at 90°C, and loaded onto a Hybond nitrocellulose membrane (Amersham) using a slot blot manifold (Bio-Rad). The membranes were hybridized to mCherry or EGFP probes to estimate the amount of reporter plasmid taken up by the cells. The probes were generated by PCR using 5′-GGATCCCACAACCATGGTGAGCAAGGGC-3′ and 5′-GCGGCCGCTACTTGTACAGCT-3′ for mCherry and 5′-GCTAGCCACAACCATGGTGAGCAAGGGC-3′ and 5′-GCTAGCTTACTTGTACAGCTCGTCCATGCCG-3′ for EGFP. Transfection efficiencies were normalized to plasmid reporter uptake.

### Real-time qRT-PCR.

Total RNA was extracted with the RNeasy kit (Qiagen) and reverse transcribed using SuperScriptIII reverse transcriptase (RT) (Life Technologies). Real-time quantitative RT-PCR (qRT-PCR) was performed on 50 to 100 ng cDNA using TaqMan Gene Expression Assays for *ACTB* (Hs99999903_m1) and *PPP1R12C* (Hs01085952_m1) and TaqMan Universal PCR master mix (Life Technologies). Relative expression levels were determined by the comparative threshold cycle (*C_t_*) method (34).

### Northern blot analysis.

Total RNA was extracted with the RNeasy kit (Qiagen). Ten micrograms (infected cells) or 2 μg (transiently transfected cells) of RNA was separated on a 1.2% formaldehyde-agarose gel and transferred onto a nitrocellulose membrane. The membranes were first hybridized to [α-^32^P]dCTP-labeled mCherry, PPP1R12C (exons 18 to 22), rep, or GFP probes; stripped at 65°C in 50% formamide, 2× SSC (1× SSC is 0.15 M NaCl plus 0.015 M sodium citrate); and rehybridized to a β-actin cDNA probe. The PPP1R12C and β-actin cDNA probes were generated as described by Dutheil et al. (27). The mCherry and GFP cDNA probes were generated by PCR using the primers mentioned above. The rep probe was generated by PCR on a plasmid containing a 315-bp PstI/SacI fragment from pAV2 using 5′-GGATCCTCAATTCTGATTCTCTTTG-3′ and 5′-CCCGGGGGTCCTGTATTAGAGGTCACGTG-3′. All Northern blots were analyzed with a Typhoon PhosphorImager (Molecular Dynamics) and then exposed to an X-ray film to generate high-quality images. ImageQuant TL software was used to calculate fold repression. Each average repression level is represented as the mean and standard error of the mean (SEM).

### Western blot analysis.

Forty-eight hours after transfection, cells were lysed in RIPA buffer (50 mM Tris-HCl [pH 8], 150 mM NaCl, 0.1% SDS, 1% Nonidet P-40, 0.5% sodium deoxycholate, 1× Complete protease inhibitor cocktail [Roche Applied Science]), and proteins were quantified by the bicinchoninic acid (BCA) protein assay (Pierce). For each condition, the same amount of protein (10 μg) was separated on a 15% SDS-polyacrylamide gel and transferred to a nitrocellulose membrane (Hybond-C Extra nitrocellulose; Amersham Biosciences). The membranes were blocked with 5% nonfat dry milk in Tris-buffered saline (TBS) (100 mM Tris-Cl, pH 7.5, 150 mM NaCl) containing 0.5% Tween 20 (TBS-T) for 1 h at room temperature and incubated with primary antibody overnight at 4°C. The membranes were washed in TBS-T buffer (three 15-min washes) and then incubated with secondary antibody conjugated to horseradish peroxidase for 1 h at room temperature. After three washes in TBS-T buffer, the membranes were developed using enhanced-chemiluminescence (ECL) substrate (Pico detection kit; Pierce). Using an ImageQuant LAS 4000 Biomolecular Imager and ImageQuant software (GE Healthcare Life Sciences), band densitometry was performed, and the result was normalized against the value of actin protein expression. After visualization of the desired protein, the membranes were stripped in Restore Western blot stripping buffer (Pierce) for 30 min at room temperature. The membranes were washed four times in TBS-T buffer, blocked with 5% nonfat dry milk, and then hybridized with specific antibody.

The primary antibodies used in the study were antibodies against mCherry (1:16,000 dilution in 1% bovine serum albumin [BSA] in TBS-T; Clontech; rabbit polyclonal red fluorescent protein [RFP] antibody; catalog no. 632397); GFP (1:5,000 dilution in 5% milk in TBS-T; Roche Applied Science; mouse monoclonal GFP antibody; catalog no. 11 814 460 001); AAV2 Rep proteins Rep78, Rep68, Rep52, and Rep40 (1:100 dilution in 5% nonfat dry milk–PBS-T for monoclonal antibody clone 303.9 [Progen Biotechnik catalog no. 61069] or 1:500 dilution in 1% BSA–PBS-T for monoclonal antibody clone 226-7 [Acris Antibodies catalog no. BM5012SU]); AAV-2 Rep proteins Rep78, Rep68, and Rep78-ΔN208 (1:200,000 dilution in 5% nonfat dry milk–PBS-T; rabbit polyclonal anti-N208 antibody); and actin proteins (1:10,000 dilution in 5% nonfat dry milk in PBS-T; BD Biosciences; mouse monoclonal actin antibody; catalog no. 612656). Polyclonal anti-N208 antibody was produced in rabbits immunized with the truncated Rep protein Rep78-ΔN208, containing the first N-terminal 208 amino acids of Rep78 and Rep68 (Cocalico Biologicals Inc.).

Goat anti-mouse (Jackson ImmunoResearch Laboratories; catalog no. 115-035-003) or anti-rabbit (Jackson ImmunoResearch Laboratories; catalog no. 111-036-003) secondary antibody conjugated to horseradish peroxidase was used at a dilution of 1:10,000 in 5% nonfat dry milk in PBS-T.

Each average repression level is represented as the mean and SEM.

### Immunofluorescence.

HeLa cells were grown in 24-well plates on coverslips coated with poly-l-lysine. At 50% confluence, the HeLa cells were transfected with 0.75 μg of DNA using the Lipofectamine Plus reagents (Invitrogen). The cells were fixed 48 h posttransfection in cold acetone, permeabilized with 0.1% Triton X-100 for 5 min, and blocked in 5% normal goat serum overnight. The slides were incubated with primary rabbit anti-N208 antibody (1:1,000 dilution in PBS) for 1 h at room temperature, washed in PBS, and incubated with a Cy3 goat anti-rabbit antibody (1:1,000 dilution in PBS; Jackson ImmunoResearch; catalog no. 711-165-152) for 1 h at room temperature. The slides were subsequently washed in PBS and mounted in Vectashield Mounting Medium containing DAPI (4′,6-diamidino-2-phenylindole; Vector Laboratories). Images were acquired at ×100 magnification using a Leica DMRA2 fluorescence microscope with a Hamamatsu charge-coupled-device (CCD) digital camera and analyzed with Openlab software (Improvision).

### Fluorescence-activated cell sorting.

To determine the effect of Rep on endogenous *PPP1R12C* expression, 293T cells transfected with the different Rep-IRES-GFP expression constructs were harvested 48 h posttransfection and sorted for GFP expression using the BD FACSAria II (Becton Dickinson) prior to RNA isolation.

## RESULTS

### Under permissive conditions, AAV2 infection leads to downregulation of *PPP1R12C* expression.

**FIG 1:**
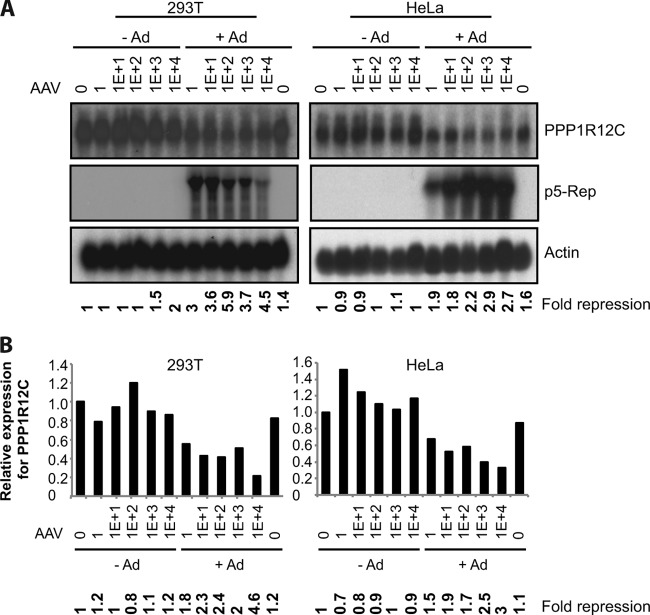
*PPP1R12C* and *rep* expression levels in AAV2-infected cells. (A) Representative example of Northern blot analysis of 293T (left) and HeLa (right) cells infected with wt AAV2 at increasing MOIs (1 to 10,000) in the absence (−Ad) and presence (+Ad) of adenovirus 48 h postinfection. Northern blots were hybridized with PPP1R12C (top), p5-Rep (middle), and β-actin (bottom) cDNA probes. Relative fold repression of *PPP1R12C* expression is indicated at the bottom. (B) Determination of *PPP1R12C* expression levels by real-time qRT-PCR in the samples shown in panel A confirms downregulation of *PPP1R12C* expression during productive AAV2 infection in two different cell lines.

To determine if AAV2 infection leads to changes in the expression levels of the target site *PPP1R12C* promoter, 293T and HeLa cells were infected with wt AAV2 using increasing MOIs in the presence and absence of adenovirus (wt Ad5). Coinfection with adenovirus leads to permissive conditions, which support efficient AAV2 replication, whereas infection with AAV2 alone represents nonpermissive conditions under which the virus can establish latency. Total RNA was extracted 48 h postinfection (p.i.), and the expression of *PPP1R12C* was analyzed by Northern blotting and real-time qRT-PCR. In the absence of adenovirus infection, expression levels of *PPP1R12C* were similar in control and wt AAV2-infected HeLa cells at all MOIs tested (Fig. 1A, top right). In 293T cells, the same tendency was observed; however, at higher MOIs, slightly lower levels of *PPP1R12C* expression were observed than in control cells (Fig. 1A, top left). In contrast to nonpermissive conditions, *PPP1R12C* expression is clearly repressed in wt AAV2- and adenovirus-coinfected cells compared to control cells, and this was observed for both cell lines (Fig. 1A, top row). Importantly, this effect does not appear to be mediated by adenovirus alone, as *PPP1R12C* expression levels were similar in control and adenovirus-infected cells. Note that the actin expression levels remained unaltered upon productive AAV2 infection. The observed repression of *PPP1R12C* expression under permissive conditions was confirmed by real-time qRT-PCR (Fig. 1B).

As the large Rep proteins, Rep78 and Rep68, are highly expressed under permissive conditions, we determined whether downregulation of *PPP1R12C* expression might be related to the presence of Rep78 or Rep68 (Rep78/Rep68) transcripts (12). As expected, the AAV2 p5 transcripts can be detected in AAV2- and adenovirus-coinfected cells, whereas Rep78/Rep68 expression cannot be detected in AAV2- or adenovirus-infected or control cells (Fig. 1A, middle row). These data indicate that a decrease in *PPP1R12C* expression might be correlated with an increase in p5 transcript levels, suggesting that the AAV2 Rep proteins can modulate *PPP1R12C* expression within its genomic locus. It is interesting that in 293T cells, despite the decrease of Rep transcript levels at higher MOIs, *PPP1R12C* expression levels remain strongly repressed.

**FIG 2:**
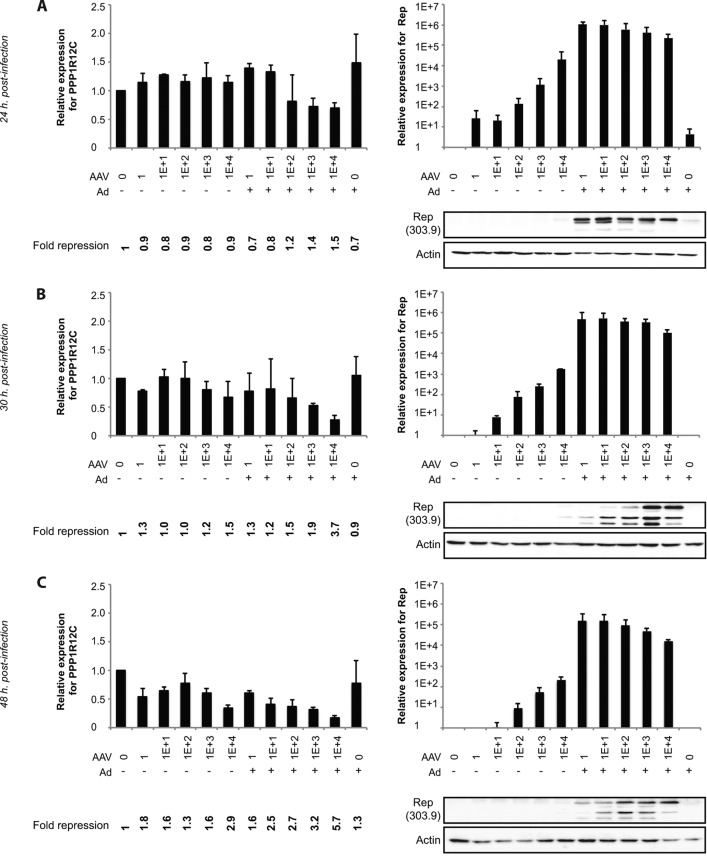
Time course of *PPP1R12C* and *rep* expression levels in AAV2-infected 293T cells. (A to C, left) Determination of *PPP1R12C* expression levels by real-time qRT-PCR in 293T cells 24 (A), 30 (B), and 48 (C) hours after AAV2 infection (at increasing MOIs in the absence and presence of adenovirus). Relative fold repression of *PPP1R12C* expression is indicated at the bottom. (A to C, top right) Determination of *rep* expression levels by real-time qRT-PCR in 293T cells 24 (A), 30 (B), and 48 (C) hours after AAV2 infection (at increasing MOIs in the absence and presence of adenovirus). Relative expression levels were determined in 2 independent infection experiments at each time point. (A to C, bottom right) Representative example of Western blot analysis of Rep expression in 293T cells 24 (A), 30 (B), and 48 (C) hours after AAV2 infection.

A potential link between downregulation of expression from the AAV2 integration target site promoter and Rep expression was further supported by a time course experiment of AAV2 and adenovirus infection in 293T cells, for which *PPP1R12C* and Rep expression levels were determined by real-time qRT-PCR. The presence of Rep proteins in AAV2- and adenovirus-coinfected cells was confirmed by Western blotting. Twenty-four hours after infection, we could observe a modest decrease in relative *PPP1R12C* expression levels in cells coinfected at a high MOI (Fig. 2A, left), which became more pronounced at later time points (Fig. 2A and B, left). At 48 h postinfection, we could also observe downregulation of expression in the absence of adenovirus. Interestingly, Rep transcripts were detected under all conditions but increased strongly when adenovirus was added to the cultures. In general, Rep transcript levels decreased as the infection progressed. Western blot analysis of the samples showed strong expression of the large Rep proteins 24 h after coinfection, increasing expression of the small Rep proteins at 30 h postinfection, and decreasing Rep protein levels at 48 h postinfection (Fig. 2A, B, and C, right).

### Both large and small Rep proteins repress *PPP1R12C* promoter activity.

**FIG 3:**
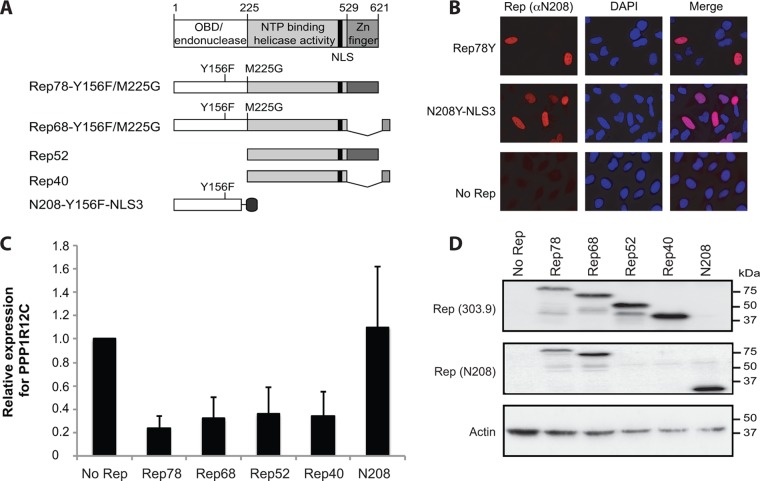
Endogenous *PPP1R12C* expression levels in Rep-transfected cells. (A) Schematic representations of the large (Rep78/Rep68), small (Rep52/Rep40), and truncated (N208) Rep proteins used for overexpression in 293T cells. At the top are shown the different functional domains present in Rep78. Nucleotide positions indicating the beginning and end of each domain are indicated above. The black bar shows the position of the NLS. Below are shown the large Rep proteins, Rep78 and Rep68, used for the analysis. Mutations were introduced to avoid endonuclease activity (Y156F) and simultaneous expression of the small Rep proteins (M225G). At the bottom are depicted the small Rep proteins (Rep52 and Rep40) and the N-terminally truncated Rep protein fused to 3 tandem repeats of the SV40 large T antigen NLS (N208-NLS3). The different Rep proteins were expressed from CMV promoter-IRES-GFP vectors. (B) Epifluorescence microscopy images of cells transfected with plasmids expressing Rep78-Y156F-N208Δ containing three copies of the SV40 large T antigen NLS and stained with DAPI and anti-Rep (α-Rep) (N208) antibody. The positive control consisted of cells transfected with a plasmid expressing full-length Rep78-Y156F; the negative control consisted of cells transfected with the CMV promoter-IRES-GFP vector. The images were taken at ×100 magnification. (C) Determination of *PPP1R12C* expression levels by real-time qRT-PCR in cells transfected with the different Rep constructs and sorted by fluorescence-activated cell sorter (FACS) for GFP expression. Relative expression levels were determined in 3 independent transfection experiments. The error bars indicate SEM. (D) Western blot analysis confirmed the presence of the respective Rep proteins in the transfected cells.

In order to test whether the observed repression is directly mediated by the viral Rep proteins, we transfected 293T cells with different Rep expression constructs. Since the chromosomal RBS and terminal resolution site (TRS) are located in the 5′ UTR of the *PPP1R12C* gene (27), it is conceivable that Rep78 and Rep68 can interfere with the *PPP1R12C* promoter activity by introducing a site-specific nick in the 5′ UTR (35, 36). Therefore, all experiments were executed using the Rep mutant RepY156F, which lacks nicking enzymatic activity (37) (Fig. 3A). Since the Rep52 and Rep40 proteins are the N-terminally truncated forms of Rep78 and Rep68, respectively, the initial methionine of Rep52 and Rep40 was mutated to a glycine to accomplish expression of the large Rep proteins only (20, 21) (Fig. 3A). We also examined whether the truncated protein containing only the first N-terminal 208 amino acids of Rep, Rep78-Y156F-ΔN208, could affect *PPP1R12C* promoter activity. The rationale for using the Rep78-Y156F-ΔN208 protein is that this truncated protein has previously been shown to be the minimal domain that can efficiently bind to both AAV2 and *PPP1R12C* RBS sequences *in vitro* while retaining its TRS endonuclease activity (30). Because Rep78-Y156F-ΔN208 lacks the bipartite NLS, which is localized in the C-terminal domain of Rep78/Rep68 (38), the C-terminal region of Rep78-Y156F-ΔN208 was fused to either one, two, or three tandem repeats of the NLS of SV40 large T antigen. In order to assess whether Rep78-Y156F-ΔN208 is effectively translocated to the nucleus, we performed immunofluorescence microscopy on cells transfected with the different NLS constructs. As shown in Fig. 3B, Rep78-Y156F-ΔN208 containing three copies of the SV40 large T antigen NLS is, like wild-type Rep78, mainly located in the nucleus. Rep78-Y156F-ΔN208 containing only one or two copies of the SV40 large T antigen NLS is predominantly located in the cytoplasm and in the perinuclear region (data not shown). All Rep variants were cloned into an IRES-GFP vector. The transfected cells were sorted for GFP expression to ensure the presence of Rep. As shown in Fig. 3C, all Rep proteins except Rep78-Y156F-ΔN208 had the ability to strongly repress endogenous *PPP1R12C* expression levels in GFP-positive cells (Fig. 3C). Western blot analysis confirmed expression of the different Rep proteins in the transfected cells (Fig. 3D).

**FIG 4:**
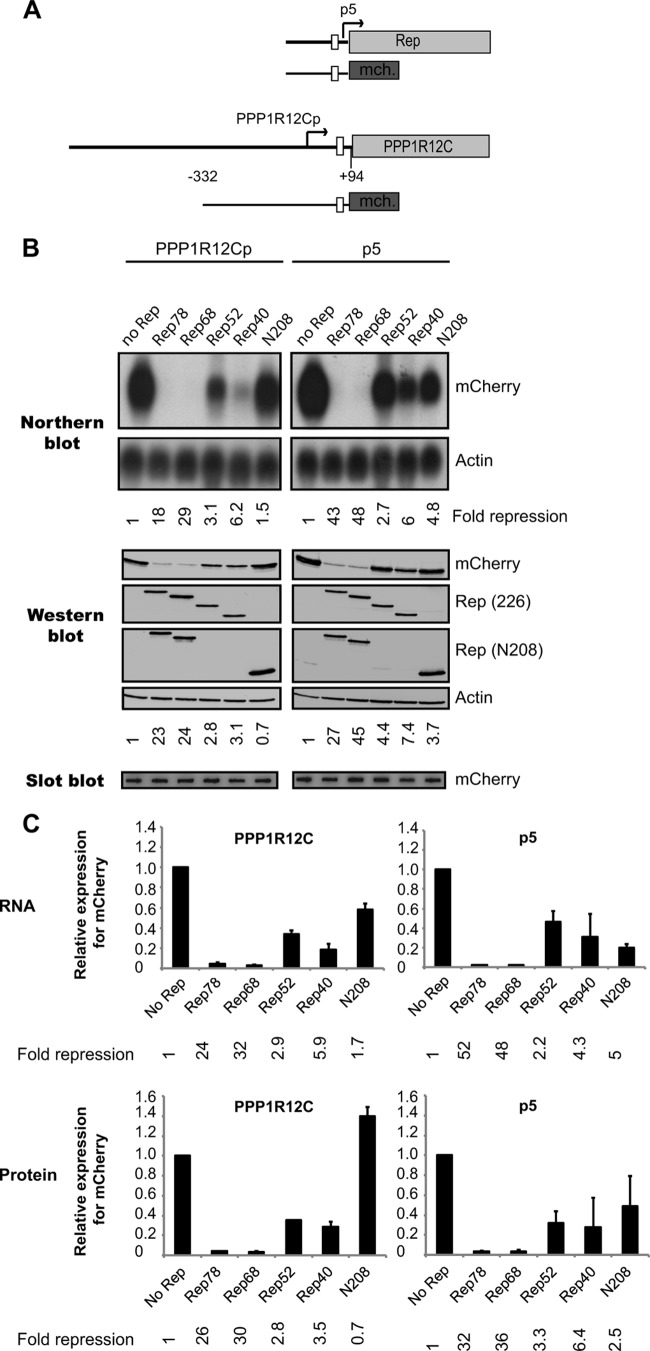
Analysis of the effects of the four AAV2 Rep proteins on *PPP1R12C* promoter (*PPP1R12C p*)- and AAV2 p5-directed gene expression. (A) Schematic representations of the AAV2 p5 and *PPP1R12C* mCherry reporter plasmids. The genomic structures of the Rep and *PPP1R12C* genes are depicted at the top. The bent arrows mark the p5 and *PPP1R12C* transcription start sites (TSS). The white boxes indicate the positions of the p5 RBS and *PPP1R12C* TRS-RBS motifs. (B) Northern blot analysis shows *PPP1R12C* (left) and p5 (right) transcription levels in 293T cells cotransfected with an mCherry reporter plasmid and the Rep-expressing plasmids shown in Fig. 3A. The blots were stripped and hybridized with a β-actin cDNA probe (lower blots). Relative fold repression of mCherry expression is indicated below the blots. Western blot analysis shows the corresponding mCherry protein levels resulting from *PPP1R12C* (left) and p5 (right) promoter activity. Rep expression was confirmed by Western blotting using the 226-7 and N208 antibodies. The blots were also incubated with a β-actin antibody, and relative fold repression was determined (indicated below the blots). Slot blot membranes hybridized to an mCherry probe show similar plasmid uptake for all experimental conditions. (C) Determination of average mCherry expression levels from the *PPP1R12C* (left) and p5 (right) promoters from 3 independent experiments by Northern and Western blot analyses. Average fold repression of mCherry expression is indicated at the bottom of each graph. The error bars indicate SEM.

In order to obtain better insight into potential mechanisms responsible for the observed Rep-mediated repression of *PPP1R12C* expression, we cotransfected Rep constructs (Fig. 3A) with reporter constructs containing the mCherry gene under the control of the *PPP1R12C* or AAV2 p5 promoter (Fig. 4A). Since Rep proteins can modulate the expression of a number of cellular and viral genes, including the cytomegalovirus (CMV) promoter (23, 24, 39), it is not possible to correct for variations in transfection efficiency by normalization to the activity of a cotransfected plasmid expressing a marker gene under the control of a viral or eukaryotic promoter. Therefore, transfection efficiencies were normalized to plasmid DNA uptake, as described previously (39). Figure 4B shows a representative example of the Northern and Western blot analyses performed. Transcript and protein levels from three independent experiments are shown in Fig. 4C. Rep78 and Rep68 strongly inhibit *PPP1R12C* promoter-driven expression at both the RNA and protein levels. In contrast to the large Rep proteins, Rep52 and Rep40 reproducibly inhibit *PPP1R12C* promoter activity, but at lower levels. Changes in RNA levels parallel those observed for protein levels. These data demonstrate that all Rep proteins have the ability to mediate repression of the *PPP1R12C* promoter despite significant differences in the levels of inhibition between Rep78/Rep68 and Rep52/Rep40. The difference in ability to repress *PPP1R12C* expression between the small and large Rep proteins was not as evident with the endogenous gene, which can be explained by a lower gene copy number than in the overexpression system. Similarly to what is shown in Fig. 3C, the level of *PPP1R12C* expression in the presence of Rep78-Y156F-ΔN208 is comparable to that detected in the absence of Rep (Fig. 4B and C). These data indicate that Rep78-Y156F-ΔN208 does not have the ability to regulate *PPP1R12C* promoter activity, suggesting that the DNA binding domain is not sufficient to inhibit *PPP1R12C* expression.

As the AAV2 p5 promoter shares common regulatory elements with the *PPP1R12C* promoter (27), the question arises as to whether the viral Rep proteins might regulate the two promoter activities by similar mechanisms. Therefore, we compared Rep's effects on the p5 and *PPP1R12C* promoters. Interestingly, all Rep proteins downregulate the AAV2 p5 promoter similarly to what we observed for the *PPP1R12C* promoter (Fig. 4B and C, right). In agreement with previously published data (19), the large Rep proteins completely repress p5 promoter activity, whereas the small Rep proteins inhibit the p5 promoter to a lesser extent. Of note, Rep78-Y156F-ΔN208 moderately represses p5 transcriptional activity, whereas the *PPP1R12C* promoter activity remains relatively unchanged in the presence of this truncated Rep protein.

### The NTP-binding motif is required for Rep52- and Rep40-mediated repression of the p5 and *PPP1R12C* promoters.

**FIG 5:**
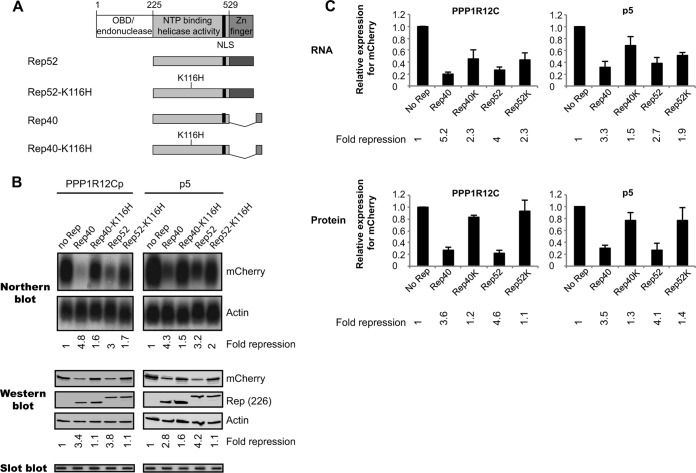
Analysis of the effects of the Rep52/Rep40 proteins and corresponding NTP-binding mutants on gene expression from the *PPP1R12C* and p5 promoters. (A) At the top are shown the different functional domains present in Rep78. Nucleotide positions indicating the beginning and end of each domain are indicated above. The black bar shows the position of the NLS. Below are shown schematic representations of the various small Rep proteins coexpressed with the *PPP1R12C* and p5 mCherry reporter plasmids. The NTP-binding mutants harbor a mutation at amino acid position 116 (K116H). (B) Northern blot analysis shows *PPP1R12C* (left) and p5 (right) transcription levels in 293T cells cotransfected with an mCherry reporter plasmid and various Rep-expressing plasmids. The blots were stripped and hybridized with a β-actin cDNA probe (lower blots). Relative fold repression of the mCherry transcription levels is indicated at the bottom of the blots. Western blot analysis shows the corresponding mCherry protein levels resulting from *PPP1R12C* (left) and p5 (right) promoter activity (top). Expression of the small Rep proteins and NTP-binding mutants was determined by hybridization with the 226-7 antibody. The blots were also incubated with a β-actin antibody, and relative fold repression was determined (indicated below the blots). Slot blot membranes hybridized to an mCherry probe show similar plasmid uptake for all experimental conditions. (C) Determination of average mCherry expression levels from the *PPP1R12C* (left) and p5 (right) promoters from 3 independent experiments by Northern and Western blot analyses. Average fold mCherry repression is indicated at the bottom of each graph. The error bars indicate SEM.

Previous studies established that a residue within the NTP-binding domain of Rep78 (K340) is critical for the negative regulation of the p19, HIV LTR, and HPV18 URR promoters (21), raising the hypothesis that the same residue might be required for *PPP1R12C* promoter repression. As Rep52 and Rep40 mainly consist of an NTP-binding domain with ATPase and helicase activities (Fig. 5A), we used the smaller Rep proteins to determine whether mutations in the NTP-binding motif would have an effect on *PPP1R12C* and p5 promoter activities. To address this, wild-type or mutant Rep proteins were cotransfected with the *PPP1R12C* or p5 reporter construct. Northern and Western blot analyses were performed to determine RNA and protein expression levels, respectively (Fig. 5B). Transcript and protein levels from 3 independent experiments are shown in Fig. 5C. Similar to the data presented in Fig. 4B and C, wt Rep52 and wt Rep40 moderately inhibit the *PPP1R12C* and p5 promoter activity, as seen for RNA and protein levels (Fig. 5B and C). In contrast to the wt Rep proteins, the mutant Rep52-K116H and Rep40-K116H proteins, which harbor a mutation that corresponds to the K340H mutation in the large Rep proteins, have no clear effect on *PPP1R12C* and p5 promoter activity (Fig. 5B and C). The relief of repression by the NTP-binding mutants is not due to differences in protein expression levels, as they are similar for all Rep constructs, or even slightly higher for the Rep40-K116H mutant than for the corresponding wt protein, as indicated by Western blotting. In sum, these data demonstrate that Rep52 and Rep40 inhibit the *PPP1R12C* and p5 promoter activities in a similar manner and that this repression requires the consensus NTP-binding motif.

### The RBS and the NTP-binding motif are both required for Rep78/Rep68-mediated repression of the p5 and *PPP1R12C* promoters.

**FIG 6:**
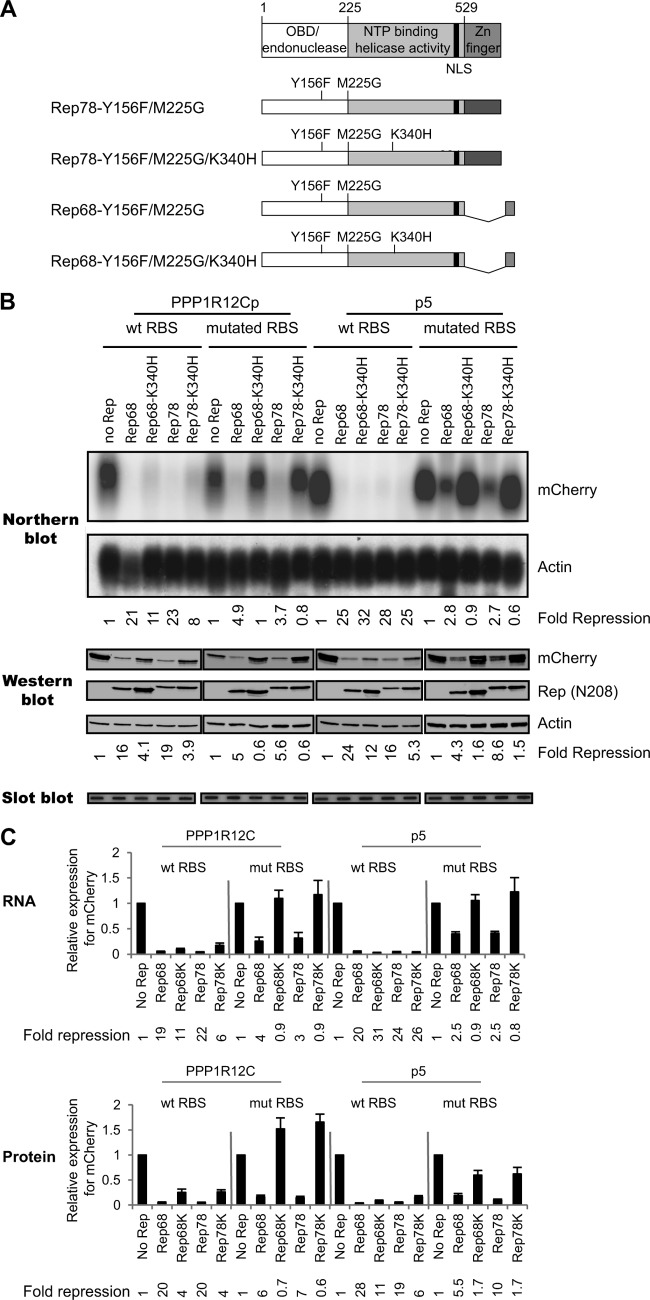
Analysis of the effects of the Rep78/Rep68 proteins and corresponding NTP-binding mutants on gene expression from wild-type and RBS mutant *PPP1R12C* and p5 promoters. (A) At the top are shown the different functional domains present in Rep78. Nucleotide positions indicating the beginning and end of each domain are shown above. The black bar shows the position of the NLS. Below are shown schematic representations of the various large Rep proteins coexpressed with the *PPP1R12C*p and p5 mCherry reporter plasmids. All Rep proteins lack endonuclease activity and contain mutations that avoid expression of the small Rep proteins (M225G). The NTP-binding mutants harbor a mutation at amino acid position 340 (K340H). (B) Northern blot analysis shows *PPP1R12C* (left) and p5 (right) transcription levels in 293T cells cotransfected with an mCherry reporter plasmid and various Rep-expressing plasmids. Rep-mediated repression was analyzed on *PPP1R12C*p and p5 reporter plasmids with unaltered, as well as mutated, RBS sequences that abolish Rep binding. The blots were stripped and hybridized with a β-actin cDNA probe (lower blots). Relative fold repression of mCherry expression is indicated at the bottom of the blots. Western blot analysis shows the corresponding mCherry protein levels resulting from *PPP1R12C* (left) and p5 (right) promoter activity (top). Expression of the large Rep proteins and corresponding NTP-binding mutants was determined by hybridization with the N208 antibody. The blots were also incubated with a β-actin antibody, and relative fold repression was determined (indicated below the blots). Slot blot membranes hybridized to an mCherry probe show similar plasmid uptake for all experimental conditions. (C) Determination of average mCherry expression levels from the *PPP1R12C* (left) and p5 (right) promoters from 3 independent experiments by Northern and Western blot analyses. Average fold repression of mCherry expression is indicated at the bottom of each graph. The error bars indicate SEM.

We next investigated the mechanism by which Rep78 and Rep68 mediate *PPP1R12C* repression. Since the AAV2 Rep78 and Rep68 proteins have the ability to bind to the RBS located within the 5′ UTR of the *PPP1R12C* gene (36), we hypothesized that, as observed for the repression of the p5 promoter (20), direct interaction of Rep with the RBS might also be involved in the inhibition of *PPP1R12C*. To address this, we used a reporter construct containing a mutation within the RBS that abolishes Rep binding to the RBS in the *PPP1R12C* promoter (36). We introduced the same mutation in the p5 RBS. Transcript and protein levels from 3 independent experiments were quantitated (Fig. 6C), and a representative example is shown in Fig. 6B. Similarly to what is shown in Fig. 4, Rep78- and Rep68-expressing plasmids strongly repress *PPP1R12C* and p5 promoter activities (Fig. 6B and C). Compared to the wt promoters, the mutation within the RBS motif reduces the ability of Rep78 and Rep68 to decrease the levels of *PPP1R12C* and p5 promoter transcripts (Fig. 6B and C). Although Rep's repressive effect on the mutant promoter is partially relieved, it is still clearly present. These data indicate that a direct interaction of Rep with the RBS is not sufficient to direct Rep-mediated repression of the *PPP1R12C* promoter, suggesting the existence of at least one additional mechanism at the basis of the observed phenomenon. It has previously been shown that Rep78 and Rep68 inhibit transcription from the p5 promoter by two different mechanisms. The first mechanism requires direct interaction of Rep with the RBS, while the second mechanism depends on the presence of a functional NTP-binding motif (20). We therefore tested the effects of the Rep78 and Rep68 nicking- and NTP-binding-negative mutant proteins (Rep78-Y156F-K340H and Rep68-Y156F-K340H) (Fig. 6A) on the wt and mutated *PPP1R12C* and p5 promoters. As shown in Fig. 6B and C (left), in the presence of an unaltered RBS, mutant Rep78/Rep68 proteins have a moderate repressive effect on the *PPP1R12C* promoter activity. This effect can also be observed for protein levels. Interestingly, the introduction of a mutation in the NTP-binding motif did not affect the repression of p5 transcription levels, as previously observed (19); however, a change in p5 repression was clear at the protein level (Fig. 6B and C, right). These results indicate that the activity of the NTP-binding motif in Rep78/Rep68 is not sufficient to accomplish complete *PPP1R12C* and p5 promoter repression. Therefore, we investigated whether the Rep78/Rep68 NTP-binding mutant proteins in the context of the mutated *PPP1R12C* and p5 promoters would lead to the complete abolishment of Rep78/Rep68's repressive effects. In contrast to the Rep78/Rep68-expressing plasmid, the NTP-binding-negative mutant was unable to repress transcription from the *PPP1R12C* and p5 promoters containing mutations in the RBS motif (Fig. 6B and C). Our data are in agreement with previously published results on Rep68-mediated repression of the p5 promoter (20). Taken together, our data provide strong evidence that Rep78/Rep68 exerts its negative regulatory effect on the *PPP1R12C* and p5 promoters by identical mechanisms. Rep-mediated repression of the p5 and *PPP1R12C* transcriptional activities requires, in addition to the NTP-binding motif, direct binding of Rep78/Rep68 to the RBS located within the promoter.

### Rep-mediated repression of the antisense promoter requires only a functional NTP-binding motif.

We have previously reported that the *PPP1R12C* promoter displays bidirectional promoter activities (27). The ability of Rep to regulate *PPP1R12C* transcription raises the question as to whether Rep is also able to regulate the transcriptional activity of the antisense *PPP1R12C* promoter. To address this question, the wt *PPP1R12C* promoter fragment used in the PPP1R12Cp-mCherry reporter construct, which exhibits both sense and antisense promoter activities (27), was cloned into a dual-reporter construct carrying the mCherry and EGFP genes in opposite directions. The *PPP1R12C* promoter drives the expression of mCherry, while the antisense promoter drives the expression of the EGFP cDNA. In order to simultaneously compare the effects of Rep on *PPP1R12C* sense and antisense promoter activities, the EGFP cDNA was deleted from all Rep-expressing constructs.

**FIG 7:**
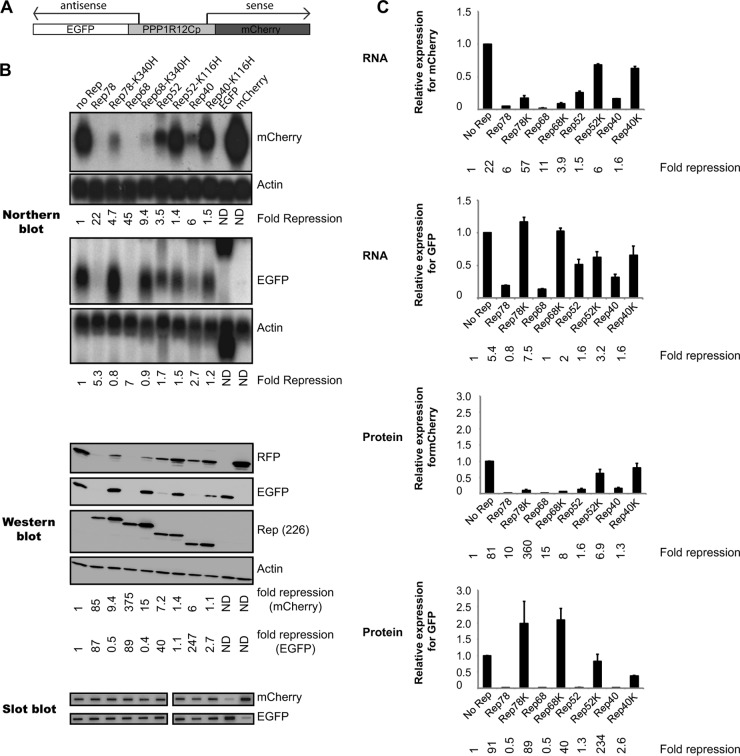
Analysis of the effects of the four Rep proteins and corresponding NTP-binding mutants on gene expression from the *PPP1R12C* sense and antisense promoters. (A) Schematic representation of the *PPP1R12C*p mCherry-GFP reporter plasmid. The Rep proteins that are coexpressed with the reporter plasmid are depicted in Fig. 5 and 6. (B) Northern blot analysis shows mCherry (top) and GFP (bottom) transcripts driven by the sense and antisense promoters, respectively. The blots were stripped and hybridized with a β-actin cDNA probe, and the relative fold repression was calculated (indicated at the bottoms of the mCherry and GFP blots). Western blot analysis shows the corresponding mCherry and GFP protein levels resulting from *PPP1R12C* (top) and antisense promoter activity (second panel from top). Expression of the Rep proteins and corresponding NTP-binding mutants was determined by hybridization with the 226-7 antibody. The blots were also incubated with a β-actin antibody, and the relative fold repression was determined (indicated below the blots). Slot blots were hybridized to an mCherry and a GFP probe to determine cellular uptake of plasmid DNA. ND, not detected. (C) Determination of average EGFP and mCherry expression levels from the *PPP1R12C* promoter from 3 independent experiments by Northern and Western blot analyses. Average fold repression of mCherry and EGFP expression is indicated at the bottom of each graph. The error bars indicate SEM.

293T cells were transiently cotransfected with the *PPP1R12C* bidirectional reporter vector and various Rep-expressing constructs. In the first set of experiments, we investigated the effects of wt and NTP-binding mutant proteins on *PPP1R12C* promoter activity in the context of the bidirectional construct (Fig. 7A). Similarly to what is shown in Fig. 4, Rep78 and Rep68 strongly repress *PPP1R12C* transcription from the bidirectional promoter, whereas Rep52 and Rep40 moderately repress the levels of *PPP1R12C* transcripts (Fig. 7B and C, mCherry). In contrast to the wt Rep proteins, Rep78/Rep68 and Rep52/Rep40 NTP-binding mutants exert different effects on the regulation of *PPP1R12C* promoter activity. Rep78 and Rep68 mutant proteins moderately repress the level of transcription, while the Rep52/Rep40 mutant proteins do not exhibit any negative regulatory effect on *PPP1R12C* promoter activity (Fig. 7B and C, mCherry). Similar observations were made for protein levels (Fig. 7B and C, mCherry).

Having validated that the mechanism by which Rep represses *PPP1R12C* transcription from the bidirectional promoter was similar to what we observed with the PPP1R12Cp-mCherry reporter construct, we next investigated the effects of Rep on the antisense promoter activity. Rep78 and Rep68 inhibit the antisense transcriptional activity by 5- and 7-fold, respectively, while Rep52 and Rep40 repress the antisense promoter by 2- and 3-fold, respectively (Fig. 7B and C, EGFP). Western blot analysis showed strong repression of the antisense promoter by all Rep proteins (Fig. 7B and C, EGFP). In contrast to the wt Rep proteins, all Rep NTP-binding mutants were unable to repress transcription from the antisense promoter (Fig. 7B and C, EGFP). This observation was also made for protein levels (Fig. 7B and C, EGFP).

Altogether, these results highlight the fact that, even though all four Rep proteins inhibit transcription from both promoters, there is a major difference in Rep78/Rep68-mediated transcriptional repression of the sense and antisense promoters. While repression of the *PPP1R12C* promoter requires the NTP-binding motif in the central domain of the Rep proteins, as well as direct interaction with the RBS, only the NTP-binding motif appears to be required for Rep78/Rep68-mediated inhibition of the antisense promoter. With regard to the small Rep proteins, our results suggest that a similar mechanism is involved in Rep52/Rep40-mediated repression of both *PPP1R12C* and antisense promoter activities and that this repression is dependent on the presence of a functional NTP-binding motif.

## DISCUSSION

The AAV Rep proteins are multifunctional proteins with the ability to regulate expression from cellular and viral promoters, including the three AAV promoters p5, p19, and p40 (7). The replication phase of the AAV life cycle is strongly dependent on controlled expression of AAV, as well as helper virus proteins, and Rep's role in this regulation has been well characterized (40). However, much less is known about a potential role for Rep in the regulation of cellular proteins that are involved in the different aspects of the AAV life cycle. In particular, it is not known if Rep has the ability to control the expression of the gene in which it integrates to establish latency. In order to gain insight into a potential additional regulatory role for Rep, we investigated if AAV2 infection and Rep expression influence the *PPP1R12C* promoter activity and compared the effect to Rep-mediated repression of the p5 promoter.

We have shown that AAV2 and adenovirus coinfection and the associated increase in Rep expression lead to a decrease in *PPP1R12C* expression levels. The observed infection-induced *PPP1R12C* repression appears to be different in 293T and HeLa cells in that the observed repression can be seen in 293T cells infected with AAV2 at high MOIs, a condition under which Rep transcripts cannot be detected by Northern blotting. However, the presence of E1A and E1B in 293T cells leads to Rep levels that are sufficient to support limited replication in the absence of helper virus (17, 41) and is thus expected to also have an effect on *PPP1R12C* transcription. An additional observation we made in 293T cells is that the level of repression in the presence of adenovirus appears to go up with increasing MOIs despite declining Rep transcripts. This decrease in Rep expression was not observed in HeLa cells and could be due to differences in AAV replication levels and autoregulatory feedback loops (41). The presence of Rep transcripts in the absence of adenovirus infection and downregulation of Rep expression over time were confirmed in the time course infection experiments in 293T cells. In summary, we observed that under permissive conditions AAV2 infection causes downregulation of *PPP1R12C* expression, which is potentially mediated by Rep but not necessarily dependent on high levels of Rep transcription.

**FIG 8:**
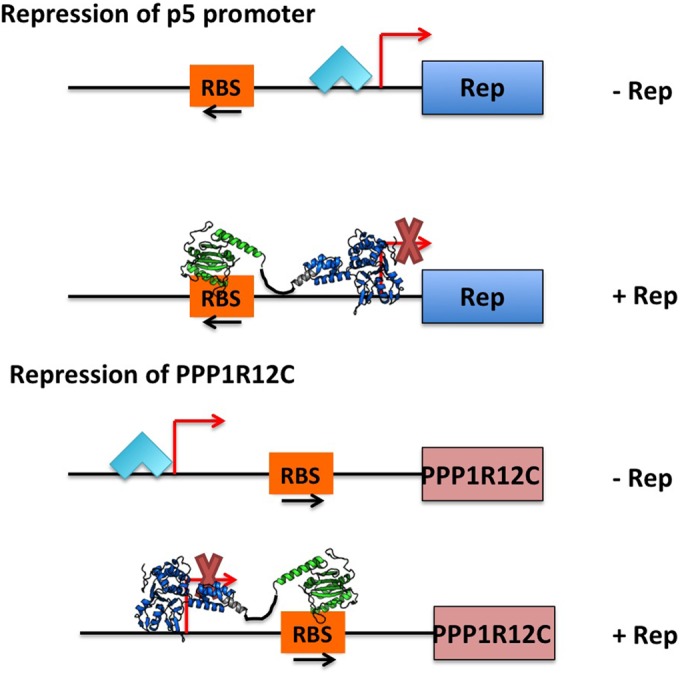
Model of Rep-mediated repression of p5 and integration target site promoters as mediated through OBD-RBS interactions. (Top) Rep78-Rep68-mediated transcriptional repression of the p5 promoter. (Bottom) Rep78-Rep68-mediated transcriptional repression of the *PPP1R12C* sense promoter. The light-blue arrowheads depict transcription factor binding. The red arrows indicate the positions of the transcriptional start sites. The black arrows indicate the directions of the 5′-GCTC-3′ repeats in the RBS. Ribbon representations of the Rep68 OBD (green), interdomain linkers (black), and NTPase domains (blue) depict RBS binding and upstream positioning of the C-terminal SF3 helicase domain, which interferes with the start of transcription and/or binding of transcriptional activators to the promoter.

Transfection-based experiments directly showed that the observed effect is indeed mediated by Rep. In fact, we could show that all four Rep proteins mediate repression of the endogenous gene, as well as the *PPP1R12C* reporter construct, and that this activity is directed by mechanisms similar to those employed by the Rep proteins for the downregulation of the p5 promoter. As was seen for repression of p5 transcription (19, 21), the large Rep proteins display a stronger repressive effect on the *PPP1R12C* promoter reporter constructs than the small Rep proteins (19, 21). This difference could be attributed to the N-terminal DNA binding domain present in Rep78 and Rep68; however, this domain by itself showed a decreased ability to suppress the *PPP1R12C* promoter compared to the full-length large Rep proteins. It is likely that the Rep78/Rep68-induced repression is dependent on the final Rep78/Rep68 oligomeric complex that assembles on the p5 and *PPP1R12C* promoter sites. This event is mediated through specific DNA binding dependent on the presence of the origin binding domain (OBD), interdomain linker, and helicase domain (42). Evidence from structural and biochemical experiments suggests that the assembly of Rep78/Rep68 complexes on RBS-containing DNA sites is both directional and highly cooperative (unpublished data). The X-ray structure of the OBD-RBS complex shows that the Rep molecules bind to the RBS positions in such a way that the C-terminal helicase domain is oriented upstream of the 5′-GCTC-3′ repeats, where it interacts with DNA nonspecifically (43). This model is supported by biochemical data showing that the affinity of Rep68 for DNA with only the minimal RBS site is significantly lower than for sites that also include upstream sequences (32). In addition, footprinting experiments have shown that Rep68 protects regions upstream of the RBS sequence (32). Taken together, it appears that binding of Rep78/Rep68 to RBS DNA sites is a highly cooperative event that requires the participation of all structural domains. Our model proposes that the repression mechanism is a direct effect of Rep78/Rep68 either blocking the start of transcription and/or binding of transcriptional activators in both the p5 and the *PPP1R12C* promoters (Fig. 8). The different directionality of the 5′-GCTC-3′ repeats in the RBS of the p5 versus *PPP1R12C* promoter allows physical interference with the initiation of transcription despite their different positions with respect to the transcriptional start site (Fig. 8). We hypothesize that the K340H mutation may have an effect on DNA affinity or complex formation, explaining the observed lower level of repression in the context of the large Rep proteins and the p5 and sense *PPP1R12C* promoter. However, we cannot exclude the possibility that an additional mechanism that is independent of binding to the RBS but requires interactions with the NTPase domain and cellular proteins also plays a role in Rep78/Rep68-mediated repression.

Our data suggest that this mechanism is likely also responsible for Rep78/Rep68-mediated repression of the antisense promoter, which lacks the RBS and, similar to what was observed for other heterologous promoters (21), requires only an intact NTP-binding motif. The small Rep proteins, which lack the OBD, might exploit the aforementioned interactions with host cell proteins in order to mediate repression of the p5 and *PPP1R12C* sense and antisense promoters (Fig. 5 and 7). In addition, nonspecific DNA binding activities of these proteins may also interfere with the binding activities of proteins from the transcription machinery by masking binding sites; however, further studies need to be performed to answer this question.

In summary, in addition to direct interactions with the promoter site, transcriptional repression of both viral p5 and cellular target site promoters may rely on interactions of Rep with factors of the transcription machinery. In fact, DNA-protein interactions, as well as protein-protein interactions, have also been described for Rep-mediated repression of the AdMLP (22). The close proximity of the Rep binding site to the TATA element in AdMLP and the observed interactions of Rep with TATA-binding protein (TBP) have provoked the hypothesis that Rep could repress gene expression by interfering with the formation of the RNA polymerase II preinitiation complex (22).

It could be hypothesized that Rep recruits factors that directly act on RNA polymerase II or exert their function by manipulating the chromatin environment (44). Indeed, several Rep-interacting partners have been identified, among which several proteins are involved in transcriptional regulation: Sp1 (45, 46), high-mobility group 1 (HMG1) nonhistone proteins (47), putative protein kinase PKX and protein kinase A (48, 49), and transcriptional coactivator PC4 (50, 51). The last is an ideal candidate, as Rep-PC4 interactions have been shown to be dependent on Rep's NTPase domain; however, preliminary knockdown experiments in our laboratory did not affect Rep-mediated repression of the *PPP1R12C* promoter (data not shown).

It is interesting to speculate that AAV2 has adapted to integrate into a chromosomal site, which appears to be regulated by protein complexes that also direct viral gene expression. In fact, once integrated, the virus may exploit these regulatory mechanisms in order to silence the integration site and associated provirus to maintain latency. Furthermore, the observation that the 5′ end of the provirus, containing the promoter region of recombinant or wt AAV2, is usually found in the 5′-3′ transcriptional direction of the *PPP1R12C* gene (52) indicates that these transcriptional protein complexes may also be involved in the formation and positioning of the preintegration complex. Future work directed at identifying Rep's binding partners involved in transcriptional regulation might therefore also shed light on the intricacies of a unique viral integration mechanism.
